# Secretory phospholipase A_2_ modified HDL rapidly and potently suppresses platelet activation

**DOI:** 10.1038/s41598-017-08136-1

**Published:** 2017-08-14

**Authors:** Sanja Curcic, Michael Holzer, Lisa Pasterk, Eva Knuplez, Thomas O. Eichmann, Saša Frank, Robert Zimmermann, Rudolf Schicho, Akos Heinemann, Gunther Marsche

**Affiliations:** 1 Institute of Experimental and Clinical Pharmacology, Medical University of Graz, Graz, Austria; 2 Institute of Molecular Biosciences, University of Graz, Graz, Austria; 3 Institute of Molecular Biology and Biochemistry, Medical University Graz, Graz, Austria; 4 BioTechMed, Graz, Austria

## Abstract

Levels of secretory phospholipases A_2_ (sPLA_2_) highly increase under acute and chronic inflammatory conditions. sPLA_2_ is mainly associated with high-density lipoproteins (HDL) and generates bioactive lysophospholipids implicated in acute and chronic inflammatory processes. Unexpectedly, pharmacological inhibition of sPLA_2_ in patients with acute coronary syndrome was associated with an increased risk of myocardial infarction and stroke. Given that platelets are key players in thrombosis and inflammation, we hypothesized that sPLA_2_-induced hydrolysis of HDL-associated phospholipids (sPLA_2_-HDL) generates modified HDL particles that affect platelet function. We observed that sPLA_2_-HDL potently and rapidly inhibited platelet aggregation induced by several agonists, P-selectin expression, GPIIb/IIIa activation and superoxide production, whereas native HDL showed little effects. sPLA_2_-HDL suppressed the agonist-induced rise of intracellular Ca^2+^ levels and phosphorylation of Akt and ERK1/2, which trigger key steps in promoting platelet activation. Importantly, sPLA_2_ in the absence of HDL showed no effects, whereas enrichment of HDL with lysophosphatidylcholines containing saturated fatty acids (the main sPLA_2_ products) mimicked sPLA_2_-HDL activities. Our findings suggest that sPLA_2_ generates lysophosphatidylcholine-enriched HDL particles that modulate platelet function under inflammatory conditions.

## Introduction

Secretory phospholipases A_2_ (sPLA_2_) are members of the phospholipase A_2_ family of enzymes which hydrolyze the sn-2 ester bond in phospholipids, generating nonesterified free fatty acids and lysophospholipids. Levels of sPLA_2_ type IIA and to a lesser extent sPLA_2_ type V highly increase during the acute phase response^1^. Epidemiologic studies showed an association between elevated sPLA_2_ activity and several inflammatory diseases^2–6^. Thus, a great effort has been devoted to developing sPLA_2_ inhibitors as new agents to treat inflammatory diseases. Of particular interest, clinical trials of sPLA_2_ inhibitors in the therapy of acute coronary syndrome, sepsis and rheumatoid arthritis, failed to prove sPLA_2_ inhibition as a promising therapeutic strategy^7–9^. Unexpectedly, pharmacological inhibition of sPLA_2_ was associated with a 60% increased risk of myocardial infarction and stroke in the VISTA-16 clinical trial^7^. A subsequent Mendelian randomization study confirmed that sPLA_2_ was unlikely to be causal in the development of atherosclerosis^10^. In addition, a recent study reported increased atherosclerosis in group X sPLA_2_-deficient mice, raising the possibility that not all sPLA_2_ types drive inflammation but that certain sPLA_2_ subtypes may be even atheroprotective and anti-inflammatory^11^.

Under acute inflammatory conditions sPLA_2_-IIA is mainly associated with high-density lipoprotein (HDL), the principal plasma carrier of phospholipids and the major substrate for sPLA_2_^12, 13^. Furthermore, sPLA_2_ types V and X, found in human and mouse atherosclerotic plaques, are highly efficient in hydrolyzing lipoprotein-associated phospholipids^2^. Inflammation and especially acute phase conditions can significantly influence metabolism of HDL and consequently its protein and lipid composition^14^. One of the characteristics of acute-phase HDL is an elevated content of lysophosphatidylcholines (LPCs), generated from strongly increased sPLA_2_ activity^15^. In patients with sepsis plasma sPLA_2_ was almost completely associated with HDL^12^, which are the major source of phospholipids in plasma. In studies using a mouse model overexpressing human sPLA_2_-IIA, plasma levels of HDL were markedly decreased, which was accompanied by reduction in HDL particle size, suggesting that HDL is a principal substrate for sPLA_2_^13, 16^. In addition, recombinant sPLA_2_-IIA and V have been shown to be more active on isolated HDL in comparison to other lipoproteins, such as LDL^17^.

Platelets have a key role in arterial thrombosis, the most common cause of myocardial infarction and stroke^18^. Accumulating evidence suggests that inflammatory responses are linked to increased platelet activation in the bloodstream of patients with systemic inflammation and sepsis^19^. It is well known that platelets substantially contribute to sepsis complications such as disseminated intravascular coagulation, thrombotic microangiopathy and multiple organ failure^20^. Platelet function has been shown to be very sensitive to the influence of plasma lipoproteins and cholesterol levels^21, 22^. However, very little is known about the effects of inflammation-induced changes in HDL composition (especially lysophospholipid enrichment) on HDL-platelet interaction and functional responses of platelets. Pharmacological inhibition of sPLA_2_ resulted in increased incidence of myocardial infarction and stroke^7^, raising the possibility that increased sPLA_2_ activity during inflammation might modulate platelet activity. Based on these observations, we tested whether sPLA_2_-induced hydrolysis of HDL-associated phospholipids generates particles that affect platelet functionality. Our findings suggest sPLA_2_-modified HDL particles potently suppress agonist induced platelet activation.

## Results

### sPLA_2_-modified HDL rapidly suppresses agonist induced platelet aggregation

In order to generate sPLA_2_-modified HDL, native HDL (nHDL) isolated from plasma of healthy human volunteers was treated with human recombinant sPLA_2_ type V. The reaction was stopped by addition of the sPLA_2_ type V inhibitor varespladib. Treatment of HDL with sPLA_2_ for 90 min (sPLA_2_-HDL-low) and overnight treatment (sPLA_2_-HDL-high) hydrolyzed ~30–40% and 70–80% of HDL-associated phosphatidylcholines, respectively (Supplementary Figure 1).

**Figure 1:**
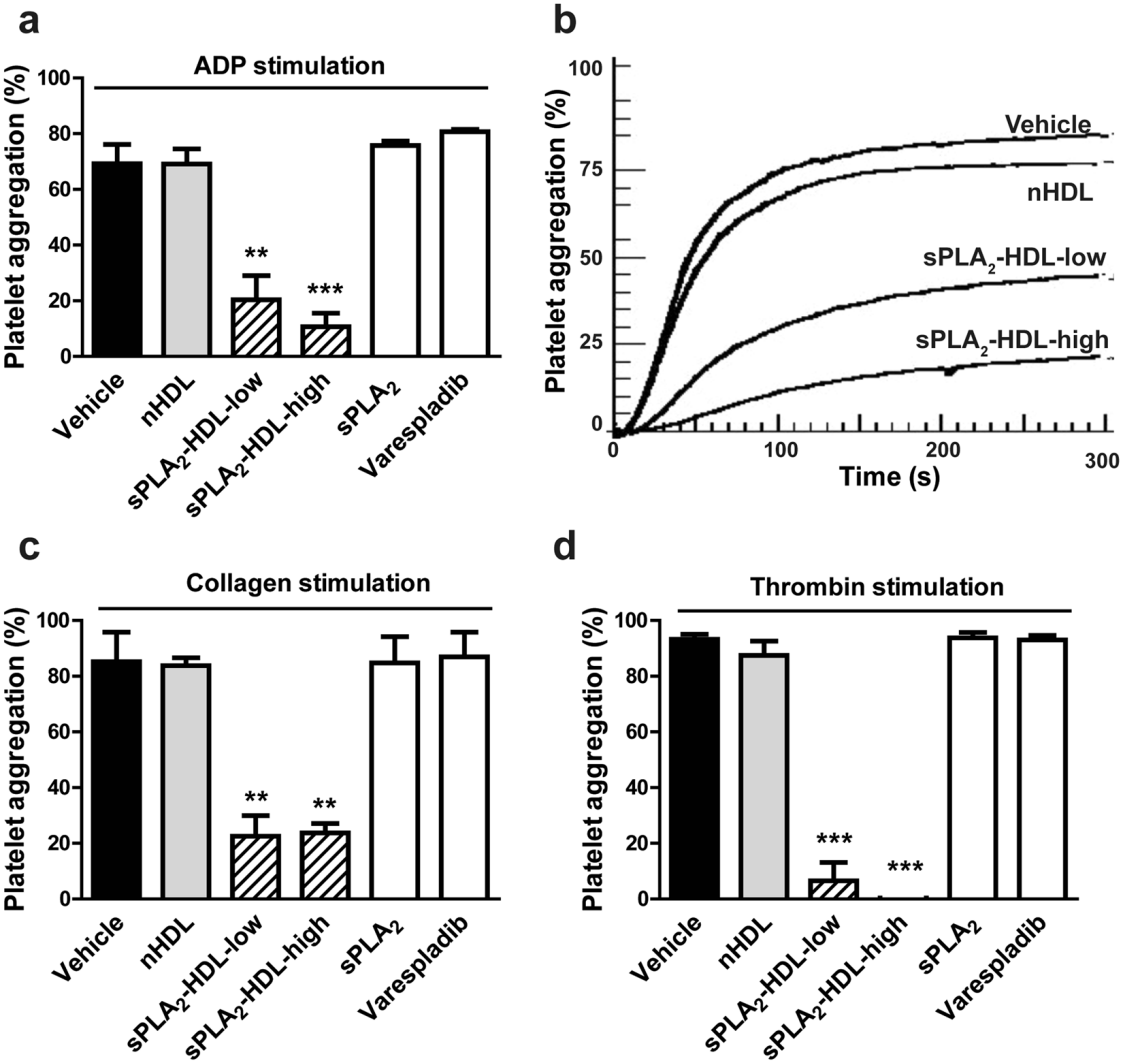
sPLA_2_-treated HDL inhibits ADP-, thrombin- and collagen-induced platelet aggregation. HDL was treated with sPLA_2_ for either 90 min (sPLA_2_-HDL-low) or overnight (sPLA_2-_HDL-high) in order to hydrolyse HDL-associated phospholipids. Platelets were preincubated with vehicle, native HDL (nHDL, 50 µg/mL), sPLA_2_-HDL-low (50 µg/mL), sPLA_2_-HDL-high (50 µg/mL), sPLA_2_ or varespladib. Subsequently, platelets were stimulated with (**a**,**b**) ADP (**c**) collagen or (**d**) thrombin in concentrations which induced 70–90% aggregation in vehicle-stimulated platelets. (**a**,**c**,**d**) Values are expressed as % of maximal platelet aggregation. Results are shown as mean ± SEM (n = 3–5). Statistical significance was assessed by one-way ANOVA followed by Dunnett’s post hoc test. **p < 0.01, ***p < 0.001 versus vehicle. (**b**) Time course of one representative recording of ADP-induced aggregation is shown.

**Figure 2:**
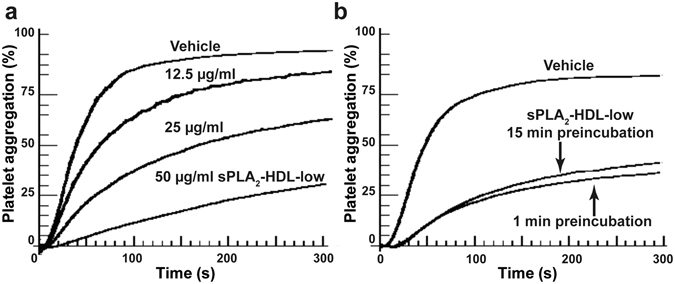
Concentration and time dependence of sPLA_2_-HDL-mediated inhibition of platelet aggregation. (**a**) Platelets were pretreated with vehicle or increasing concentrations of sPLA_2_-HDL-low (0 up to 50 µg/mL). (**b**) Platelets were preincubated with vehicle or sPLA_2_-HDL-low (50 µg/mL) for 1 or 15 min. Subsequently, platelets were stimulated with ADP in concentration which induced 70–90% aggregation in vehicle-stimulated platelets. Values are expressed as % of maximal platelet aggregation. One representative experiment out of three independent experiments is shown.

To test whether sPLA_2_-HDL affects platelet aggregation, platelets isolated from plasma of healthy donors were exposed to different agonists including ADP, collagen and thrombin in the presence or absence of native HDL and sPLA_2_-HDL. Strikingly, sPLA_2_-HDL potently inhibited platelet aggregation induced by ADP, collagen and thrombin, whereas sPLA_2_ in the absence of HDL and the sPLA_2_ inhibitor varespladib showed no effect (Fig. 1a–d). sPLA_2_-HDL-mediated inhibition of platelet aggregation was concentration dependent (Fig. 2a) and aggregation was significantly decreased already after one minute pre-treatment of platelets with sPLA_2_-HDL (Fig. 2b). Importantly, Annexin-V staining of phosphatidylserine surface expression confirmed that sPLA_2_-HDL did not affect platelet viability (Supplementary Figure 2). Furthermore, since it has been shown that HDL isolation procedure can have a significant impact on HDL composition^23^, we tested whether HDL isolated by a different method will have the same effects on platelets. Therefore, we isolated HDL from human plasma by dextran-sulfate precipitation^24^. We found that HDL isolated by dextran-sulfate precipitation also inhibited platelet activation after sPLA_2_-mediated modification (Supplementary Figure 3), whereas native HDL showed no effect.

### sPLA_2_-HDL inhibits P-selectin expression, GPIIb/IIIa activation and superoxide production in platelets

**Figure 3:**
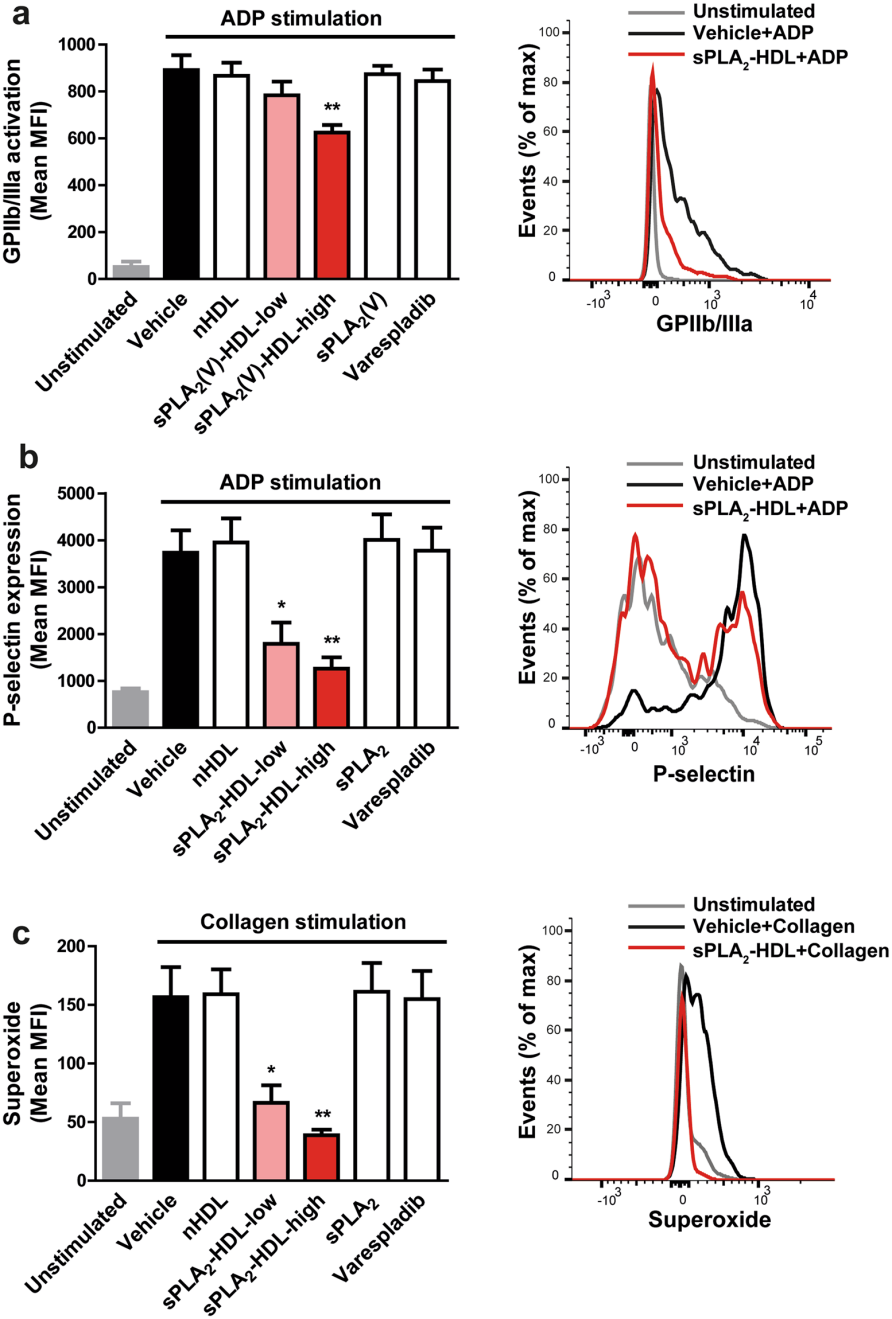
sPLA_2_-HDL inhibits P-selectin expression, GPIIb/IIIa activation and superoxide production in platelets. Platelets were pretreated with vehicle, native HDL (nHDL, 50 µg/mL), sPLA_2_-HDL-low (50 µg/mL), sPLA_2_-HDL-high (50 µg/mL), sPLA_2_ or varespladib. (**a**) For GPIIb/IIIa activation platelets were stimulated with ADP (3 µM). (**b**) Surface expression of P-selectin was induced with ADP (3 µM) in the presence of cytochalasin B (5 µg/mL) (**c**) Superoxide production was induced with 5 µg/mL collagen. (**a**–**c**) GPIIb/IIIa activation, P-selectin expression and superoxide production were measured by flow cytometry. Bar graphs on the left represent mean fluorescence intensity (MFI). Results are shown as mean ± SEM (n = 4–6). Representative flow cytometry histograms of platelets pretreated with sPLA_2_-HDL-high are displayed on the right. Statistical significance was assessed by one-way ANOVA followed by Dunnett’s post hoc test. *p < 0.05, **p < 0.01 versus vehicle-pretreated and (**a**,**b**) ADP or (**c**) collagen-stimulated platelets.

Furthermore, we assessed platelet functional responses involved in hemostatic and immune platelet functions, such as P-selectin expression, integrin activation, and reactive oxygen species production. Notably, sPLA_2_-HDL inhibited activation of the main platelet integrin GPIIb/IIIa (Fig. 3a) as well as surface exposure of the platelet activation marker P-selectin (Fig. 3b). Upon activation, platelets produce reactive oxygen species which promote their recruitment to a growing thrombus^25^. Importantly, sPLA_2_-HDL significantly attenuated superoxide anion production by platelets (Fig. 3c).

### sPLA_2_-generated lysophosphatidylcholines mediate effects of sPLA_2_-HDL on platelets

**Figure 4:**
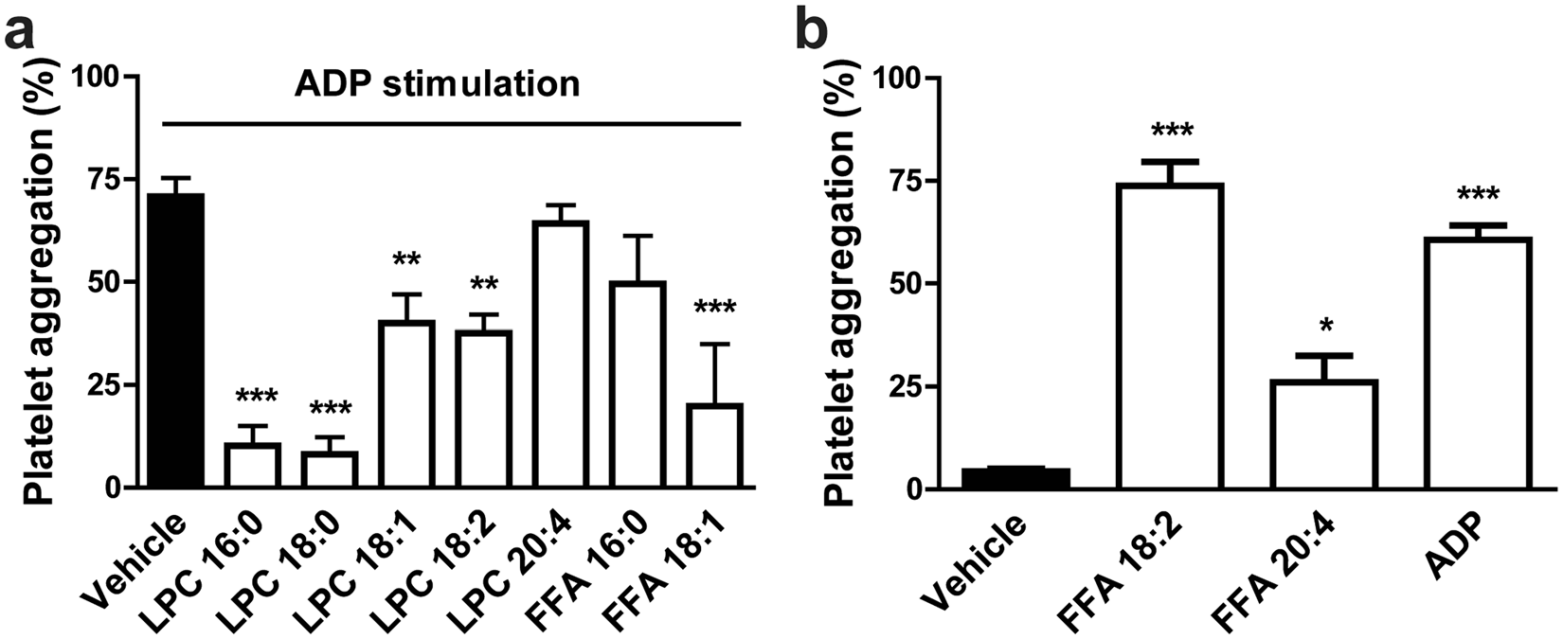
HDL-associated LPCs mediate sPLA_2_-HDL effects on platelets. (**a**) Washed platelets were preincubated with vehicle or HDL enriched with different LPCs (16:0, 18:0, 18:1, 18:2, 20:4) or FFAs (16:0, 18:1). Subsequently, platelets were stimulated with ADP inducing 70–90% aggregation in vehicle-stimulated platelets. (**b**) Platelets were stimulated with vehicle, HDL enriched with FFA 18:2, HDL enriched with FFA 20:4 or with ADP (20 µM) as a positive control and the aggregation was recorded. Values are expressed as % of maximal platelet aggregation. Results are shown as mean ± SEM (n = 3). Statistical significance was assessed by one-way ANOVA followed by Dunnett’s post hoc test. *p < 0.05, **p < 0.01, ***p < 0.001 versus vehicle.Results shown are mean ± SEM (n = 3).

In comparison to native HDL, sPLA_2_-modified HDL contains significantly higher amounts of LPCs (Supplementary Figure 1b). In order to investigate whether HDL-bound LPCs are the active moiety of sPLA_2_-HDL, native HDL was enriched with different LPC species. Saturated LPC species (16:0 and 18:0) are the most abundant LPC species in sPLA_2_-HDL and account for about 70% of total LPCs (Supplementary Figure 1b). Importantly, when enriched in HDL, LPCs mimicked the effects of sPLA_2_-HDL, with LPC 16:0 and LPC 18:0 being the most potent mediators (Fig. 4a). In addition, other LPCs species (LPC 18:1 and LPC 18:2) as well as free fatty acid (FFA) 18:1 were able to inhibit platelet aggregation, but to a much weaker extent (Fig. 4a). These results indicate that several LPCs and some FFA species contribute to the effects of sPLA_2_-HDL on platelets. In sharp contrast, FFA 18:2 and FFA 20:4 induced platelet aggregation when enriched in HDL (Fig. 4b).

### sPLA_2_-HDL suppresses agonist-induced Ca^2+^ flux and inhibits kinase phosphorylation in platelets

**Figure 5:**
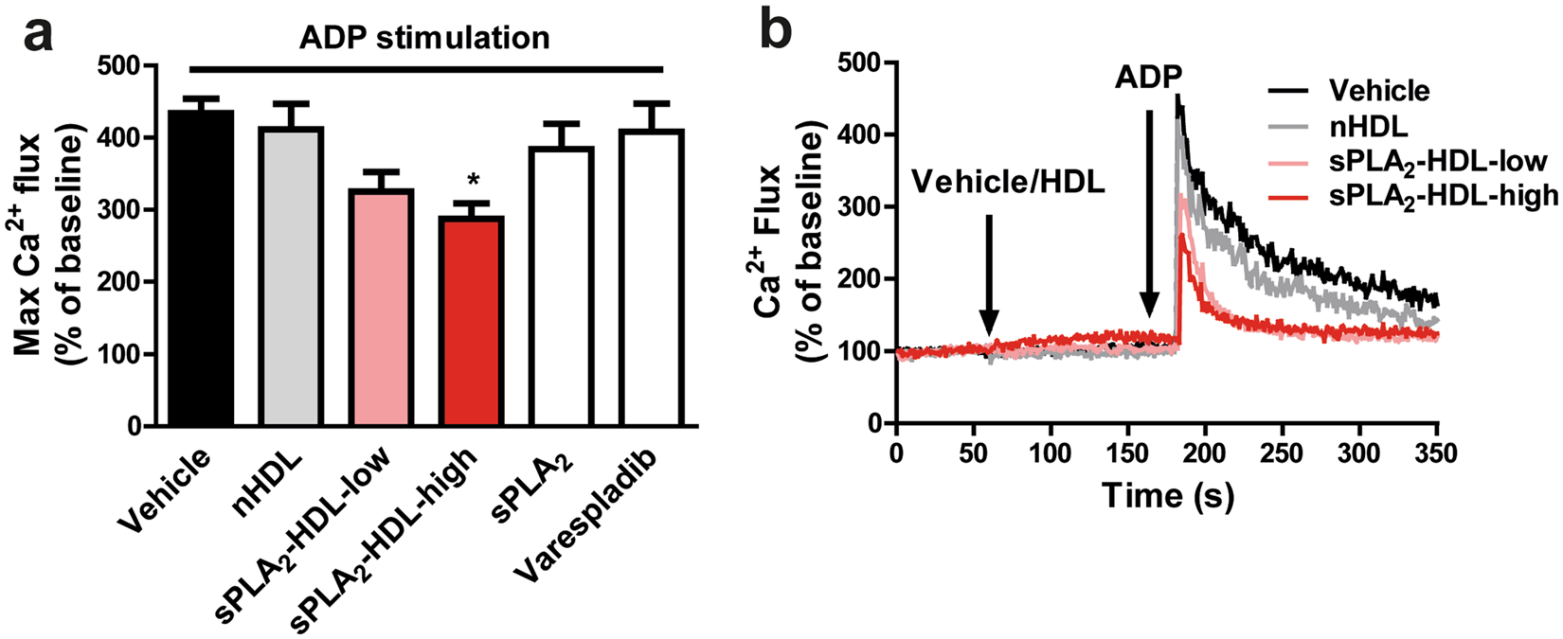
sPLA_2_-HDL inhibits Ca^2+^ flux in platelets. Baseline Ca^2+^ levels were measured by flow cytometry for 1 min and then platelets were treated either with vehicle, nHDL (50 µg/mL), sPLA_2_-HDL-low (50 µg/mL), sPLA_2_-HDL-high (50 µg/mL), sPLA_2_ or varespladib for 2 min. Ca^2+^ flux was subsequently induced with ADP as indicated by the arrow (10 µM). (**a**) Values are normalized to the baseline and expressed as maximal Ca^2+^ flux upon ADP stimulation. Results are shown as mean ± SEM (n = 4). Statistical significance was assessed by one-way ANOVA followed by Dunnett’s post hoc test. *p < 0.05 versus vehicle-pretreated and ADP-stimulated platelets. (**b**) One representative recording of Ca^2+^ flux is shown.

To gain insights into the signaling pathways involved in sPLA_2_-HDL effects on platelets, we examined intracellular signaling events such as release of Ca^2+^ (Ca^2+^ flux) and phosphorylation of protein kinases, both of which play a crucial role in platelet activation cascades. First, we assessed agonist induced changes in Ca^2+^ flux. ADP stimulation leads to a rise in intracellular Ca^2+^ in platelets, which was significantly suppressed upon sPLA_2_-HDL treatment (Fig. 5a,b).

**Figure 6:**
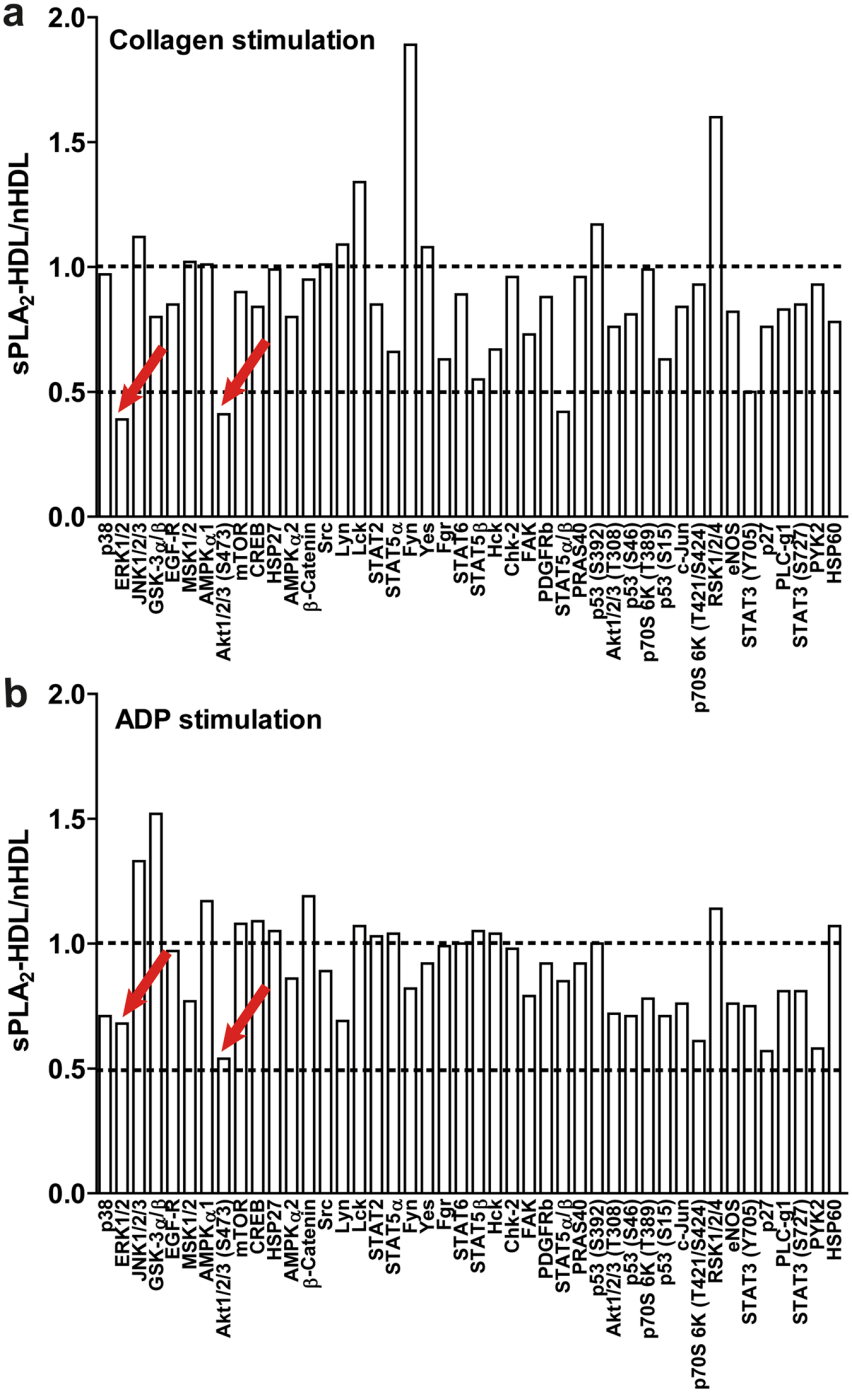
Proteome profiler analysis of nHDL and sPLA_2_-HDL-treated platelets. Platelets were preincubated with nHDL (50 µg/mL) or sPLA_2_-HDL-high (50 µg/mL) and stimulated with (**a**) ADP (10 µM) or (**b**) collagen (5 µg/mL). Kinase phosphorylation was assessed using human phospho-kinase antibody array. Values are expressed as fold increase over nHDL-treated platelets. A twofold increase/decrease in phosphorylation was considered to be significant. One representative experiment out of 3 independent experiments is shown.

**Figure 7:**
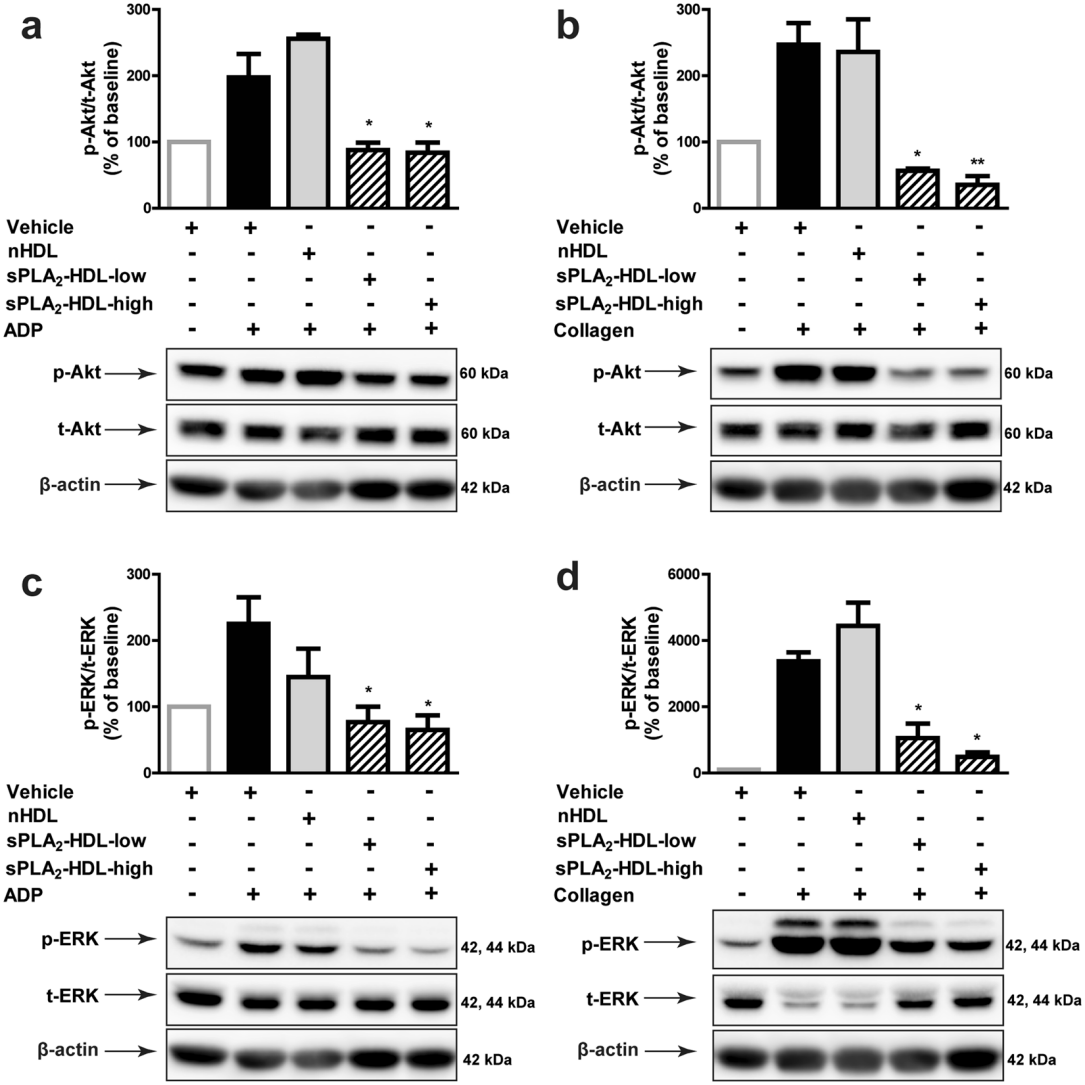
sPLA_2_-HDL inhibits Akt and ERK1/2 phosphorylation in platelets. Platelets were pretreated with vehicle, nHDL (50 µg/mL), sPLA_2_-HDL-low (50 µg/mL) or sPLA_2_-HDL-high (50 µg/mL). Subsequently cells were stimulated with (**a**,**c**) ADP (10 µM) or (**b**,**d**) collagen (5 µg/mL). (**a**,**b**) Akt (Ser473) and (**c**,**d**) ERK1/2 phosphorylation was assessed by Western blot. β-actin was used as the loading control. Blots have been cropped from full-length blots shown in Supplementary Figure 4. ChemiDoc Touch Imaging System and ECL Blotting Substrate (both Bio-Rad, Vienna, Austria) were used to visualize protein bands. Immunoblot images were quantified using Image Lab 5.2 software (Bio-Rad). Statistical analysis of phospho-Akt (p-Akt)/total Akt (t-Akt) and phospho-ERK1/2 (p-ERK)/total ERK1/2 (t-ERK) ratios were derived from 3 independent experiments which were run under the same conditions. Vehicle treated (unstimulated) control was set as baseline and values are expressed as % over baseline. Results are shown as mean ± SEM. Statistical significance was assessed by one-way ANOVA followed by Dunnett’s post hoc test. *p < 0.05, **p < 0.01 versus vehicle-pretreated and (**a**,**c**) ADP or (**b**,**d**) collagen-stimulated platelets.

To identify sPLA_2_-HDL modulated signaling cascades, we screened the phosphorylation status of kinases (including p38 MAPK, Akt, eNOS, ERK1/2, p38 MAPK, members of the Src family kinases, GSK-3α/β, AMPK, STAT3) important in platelet activation. For that purpose, platelets were activated with the platelet agonists collagen or ADP in presence of sPLA_2_-HDL or control HDL. We observed that in comparison to control HDL-treated platelets, phosphorylation of Akt at Ser473 and ERK1/2 was significantly decreased in both collagen and ADP-stimulated platelets pre-treated with sPLA_2_-HDL (Fig. 6). Effects of sPLA_2_-HDL on Akt and ERK1/2 phosphorylation were further confirmed by Western blot analysis (Fig. 7 and Supplementary Figure 4). These data suggest that inhibition of Akt and ERK1/2 phosphorylation by sPLA_2_-HDL is involved in the observed anti-aggregatory effects. Both, phosphorylation of Akt and ERK1/2 are well known to induce platelet activation^26–28^.

## Discussion

Increasing evidence suggests that HDL exerts its cardioprotective, antithrombotic and anti-inflammatory roles via direct immunomodulatory effects on several cell types including macrophages, neutrophils and endothelial cells^29–32^. Furthermore, HDL can actively interact with platelets, which results in modulation of platelet function^21, 33–35^. Most investigations showed inhibitory effects of native HDL on platelets^22^. However, there are also studies showing no effect or even pro-aggregatory activity of native HDL^36, 37^.

Previous studies reported that agonist-induced platelet aggregation is suppressed when platelets are exposed to mildly oxidized HDL^38–40^. In line with these previous studies, we show that sPLA_2_-mediated modification of HDL does not impair its anti-aggregatory activities towards platelets, but rather generates particles with increased ability to rapidly and potently suppress platelet activation. It is well known that mild oxidation generates LPCs^41, 42^. Therefore, it is likely that the anti-aggregatory activities observed with oxidized HDL^38–40^ are mediated, at least in part, by HDL associated LPCs.

sPLA_2_-HDL inhibited platelet activation induced by a wide range of physiological agonists including ADP, collagen and thrombin in a concentration-dependent manner. Interestingly, in our experiments, native HDL showed only minimal effects and sPLA_2_ in the absence of HDL showed no effect on agonist-induced activation of platelets. Therefore, our results clearly suggest that a certain threshold of HDL-associated LPC is required to suppress platelet activation. To keep the LPC content of control HDL low, we used HDL that was rapidly isolated within a few hours by using gradient ultracentrifugation^43–45^ from blood obtained from blood donors within one day. We obtained very similar results when HDL was isolated by dextran sulfate precipitation, clearly suggesting that the rapid gradient ultracentrifugation procedure to isolate HDL does not markedly affect the LPC content of HDL. A rapid isolation procedure and usage of HDL is of critical importance, given that HDL-associated lecithin-cholesterol acyltransferase and lipoprotein-associated phospholipase A_2_ constantly produce LPC. Therefore, it is reasonable to assume that the LPC content of native HDL used in different studies can determine the anti-aggregatory activities of HDL^46–48^.

Beside aggregation, sPLA_2_-HDL inhibited other platelet functional responses such as integrin GPIIb/IIIa activation and P-selectin expression. This is of importance given that these proteins not only mediate hemostatic and prothrombotic platelet functions, but also their immune interactions, such as activation of endothelium, formation of platelet-leukocyte complexes and priming of neutrophils to produce neutrophil extracellular traps^49–51^. In addition, activated platelets produce reactive oxygens species which are involved in platelet intracellular signaling, as well as platelet recruitment and thrombus growth when released from platelets^25, 52^. Importantly, sPLA_2_-HDL was able to significantly suppress platelet superoxide anion production.

Physiological levels of LPCs in the circulation are high (around 190 µM)^53^ and can reach even mM levels in some pathophysiological states, such as hyperlipidemia^54^. LPCs have been regarded to be involved in the etiology of several chronic inflammatory diseases including autoimmune diseases and atherosclerosis^55^. On the other hand, administration of LPC significantly reduced sepsis-induced mortality in mice^56^. In line with this study, septic patients show positive association between serum LPC levels and survival^57^. Biological activities of free LPC on endothelial and immune cells have been shown in many studies^55, 58^. Although LPC might be transiently found in its free form, it is predominantly bound to albumin, other serum proteins and lipoproteins^55^. The albumin-bound fraction represents an inactive form of LPC since albumin abolishes most of LPC cellular effects^59^. Interestingly, physiological activity of lipoprotein-bound LPC is not well investigated. In comparison to low-density lipoproteins, HDL carries higher amounts of LPC^60, 61^ because HDL-associated lecithin-cholesterol acyltransferase constantly produces LPC. Moreover, HDL has the ability to efficiently remove saturated and monounsaturated LPCs from low-density lipoproteins^62^. Of particular interest, deficiency in lecithin-cholesterol acyltransferase, which is the main enzyme involved in formation of HDL-associated lysophospholipids under physiological conditions^63^, decreased LPS-neutralizing capacity of HDL and enhanced LPS-induced inflammation in mice^64^. In line with these observations, we previously showed that sPLA_2_-HDL has potent anti-inflammatory effects on neutrophils^65^, indicating that HDL-bound LPCs represent a biologically active LPC fraction.

Importantly, HDL enriched with LPC 16:0 and LPC 18:0, which are the most abundant LPC species in sPLA_2_-HDL, mimicked the effects of sPLA_2_-treated HDL on platelets. Other LPC species (LPC 18:1 and LPC 18:2) and some FFAs (FFA 18:1), showed weaker inhibitory activity when enriched in HDL. This suggests that other sPLA_2_-generated products contribute to the sPLA_2_-HDL activity on platelets. In addition, sPLA_2_-HDL carries a certain amount of pro-aggregatory mediators such as arachidonic acid^66^, which induced platelet aggregation when enriched in HDL. However, their activity seems to be completely counteracted by HDL-associated LPCs. Given that the sPLA_2_ group of enzymes comprises several subtypes with different substrate specifities^67^, various LPC and FFA species are expected to be formed und inflammatory conditions that might generate sPLA_2_-HDL with diverse effects on platelets.

Of particular interest, we observed that sPLA_2_-HDL effectively suppressed agonist-induced rise in intracellular Ca^2+^ as well as phosphorylation of Akt at Ser473 and ERK1/2. Importantly, Akt and ERK play a significant role in platelet secretory pathways leading to granule release. Additionally they are involved in regulation of integrin GPIIb/IIIa function^26–28^. ERK has been shown to phosphorylate and activate cytoplasmic phospholipase A_2_, an enzyme responsible for thromboxane A_2_ synthesis^68^. Furthermore, phosphorylation of certain kinases, such as Akt, depends on the interaction with membrane cholesterol-rich microdomains^69^. We have recently shown that sPLA_2_-HDL depicts an increased cholesterol-mobilizing capacity and disrupts membrane cholesterol-rich microdomains in neutrophils^65^. Given that changes in membrane cholesterol content highly influence platelet functionality^70^, we expect that sPLA_2_-HDL-mediated cholesterol depletion might also be involved in observed inhibitory effects on agonist-induced platelet activation.

A potential limitation of the present study is that we only assessed the impact of sPLA_2_ on HDL functionality, while under inflammatory conditions HDL particles are subjected to an array of complex proteomic and lipidomic changes that affect HDL function^60, 61^. However, our study clearly demonstrates that upon modification by sPLA_2_, HDL is transformed into a particle with a strong ability to modulate platelet activation via suppression of intracellular Ca^2+^ increase and inhibition of Akt and ERK1/2 kinase phosphorylation. Our results may, therefore, contribute to a better understanding of the role of sPLA_2_ in atherosclerosis and inflammation.

## Materials and Methods

All reagents were from Sigma (Vienna, Austria), unless otherwise specified. Human recombinant secretory phospholipase A_2_ type V was purchased from Cayman Europe (Tallin, Estonia). Varespladib was purchased from Eubio (Vienna, Austria). Fluo-3-AM was from Life Technologies (Vienna, Austria). ADP, collagen and thrombin were purchased from Probe&Go (Osburg, Germany). sn-1 LPCs (16:0, 18:0, 18:1, 18:2 and 20:4) were obtained from Avanti Polar Lipids (Birmingham, AL, USA). CellFix, FACS-Flow, Annexin V, FITC-conjugated mouse anti-human CD62P (P-selectin) antibody and FITC-conjugated mouse anti-human PAC-1 antibody were from BD Bioscience (Vienna, Austria). Antibodies against ERK1/2 (#9102), phospho-ERK1/2 (Thr202/Tyr204) (#9101), Akt (#9272), phospho-Akt (Ser 473) (#9271) were from Cell Signaling Technology (Danvers MA, USA). β-actin antibody (A5316) was from Sigma (Vienna, Austria). HRP-linked goat-anti mouse (#115-036-062) and goat-anti rabbit (#111-036-045) secondary antibodies were obtained from Jackson ImmunoResearch (West Grove, USA). Fixative solution was prepared by adding 9 mL distilled water and 30 mL FACS-Flow to 1 mL CellFix.

### Blood collection

The study was approved by the Ethics Committee of the Medical University of Graz. All volunteers signed an informed consent form in agreement with the Institutional Review Board of the Medical University of Graz. All methods were carried out in accordance with the approved guidelines. Blood was collected into tubes with 3.8% (w/v) sodium citrate and platelet rich plasma (PRP) was obtained by centrifugation at 400 × g for 20 min at RT as described^71, 72^. Subsequently, plasma was used for HDL and platelet isolation.

### HDL isolation

HDL was isolated by density gradient ultracentrifugation as described^43–45^. Plasma density was adjusted with potassium bromide to 1.24 g/mL and a two-step density gradient was generated in centrifuge tubes (16 × 76 mm, Beckman) by layering the density-adjusted plasma (1.24 g/mL) underneath a NaCl-density solution (1.063 g/mL). Tubes were sealed and centrifuged at 90,000 rpm for 4 h in a 90Ti fixed angle rotor (Beckman Instruments, Krefeld, Germany). After centrifugation, the HDL-containing band was collected and desalted via PD10 columns (GE Healthcare, Vienna, Austria) and immediately used for experiments.

### sPLA_2_ treatment of HDL

HDL was incubated in the presence of 400 ng/mL human recombinant type V sPLA_2_ in PBS containing Ca^2+^ and Mg^2+^ at 37 °C for either 90 min (low modification) or overnight (high modification) in order to hydrolyse HDL-associated phospholipids. The reaction was stopped by addition of sPLA_2_ inhibitor varespladib (1 µM).

### Lysophosphatidylcholine (LPC) and free-fatty acid (FFA) enrichment of HDL

FFAs were dissolved in ethanol and LPCs in chloroform/methanol and stored at −20 °C under argon atmosphere. Required amounts of LPC were dried under a stream of nitrogen and re-dissolved in PBS. In order to generate LPC- or FFA-enriched HDL, 1 mg/mL HDL was incubated with 0.6 mmol/L 16:0, 18:1, 18:2 or 20:4 FFA or with 0.6 mmol/L 16:0, 18:1, 18:2 or 20:4 LPC for 2 h at 37 °C. Unbound LPCs and FFAs were removed by gel filtration. Content of HDL-associated LPCs was assessed by Azwell LPC Assay Kit (Hölzl Diagnostika) and FFA content was were determined using non-esterified fatty acids kit (Diasys, Holzheim, Germany).

### Platelet preparation

Platelets from platelet rich plasma were sedimented by centrifugation (1000 × g for 15 min at RT). Subsequently, platelets washed twice with a low pH platelet wash buffer (140 mM NaCl, 10 mM NaHCO_3_, 2.5 mM KCl, 0.9 mM Na_2_HPO_4_, 2.1 mM MgCl_2_, 22 mM C_6_H_5_Na_3_O_7_, 0.055 mM glucose monohydrate and 0.35% bovine serum albumin, pH = 6.5) by centrifugation (1000 × g for 15 min at RT). The final platelet preparation was resuspended in Tyrode’s buffer (10 mM HEPES, 134 mM NaCl, 1 mM CaCl_2_, 12 mM NaHCO_3_, 2.9 mM KCl, 0.34 mM Na_2_HPO4, 1 mM MgCl_2_ and 0.055 mM glucose, pH = 7.4) and used for functional platelet assays.

### Platelet aggregation

Platelet aggregation was performed at 37 °C with constant stirring (1000 rpm) using the four-channel platelet aggregometer APACT4004 (LABiTec, Ahrensburg, Germany), which works on the principle of light transmission, as previously described^71, 73^. Platelet response to a concentration range of ADP (5–20 µM), collagen (2.5–10 µg/mL) or thrombin (0.05–0.1 U/mL) was tested before each experiment. ADP, collagen and thrombin concentrations which induced 70–90% aggregation were used for platelet stimulation. Platelets were preincubated with vehicle or HDL samples for 5 min at 37 °C. Aggregation was induced with ADP in the presence of 1 µg/mL fibrinogen, collagen or thrombin and measured for 5 min. Data were expressed as percentage of maximum light transmission, with non-stimulated washed platelets being 0% and Tyrode’s buffer 100% aggregation^71, 72^.

### P-selectin (CD62P) expression

Washed platelets resuspended in Tyrode’s buffer were preincubated with vehicle, HDL samples (50 µg/mL), sPLA_2_ (400 ng/mL) or varespladib (1 µM) for 10 min at RT. Subsequently, cells were stimulated with ADP (3 µM) in the presence of cytochalasin B (5 µg/mL) for 30 min at RT in the presence of anti-CD62P-FITC conjugated antibody. Cytochalasin B was used to facilitate translocation of P-selectin from granules to the cell surface. The samples were fixed and P-selectin upregulation was detected by flow cytometry.

### GPIIb/IIIa (PAC-1) activation

PRP was suspended in Tyrode’s buffer and pretreated with vehicle, HDL samples (50 µg/mL), sPLA_2_ (400 ng/mL) or varespladib (1 µM) for 10 min at RT. The activation of glycoprotein receptor GPIIb/IIIa was induced with ADP (3 µM) for 30 min at RT in the presence of the FITC-conjugated anti-PAC-1 antibody that recognizes a conformation-dependent determinant on the GPIIb/IIIa complex. The samples were fixed and GPIIb/IIIa activation was detected by flow cytometry.

### Superoxide production

Washed platelets (1 × 10^7^ per sample) resuspended in Tyrode’s buffer were pretreated with vehicle, HDL samples (50 µg/mL), sPLA_2_ (400 ng/mL) or varespladib (1 µM) for 10 min at 37 °C and stimulated with collagen (5 µg/mL) for 20 min, at 37 °C. Superoxide production was detected with Superoxide Detection Kit according to manufacturer’s protocol. Briefly, after the treatment platelets were washed once, resuspended in superoxide detection reagent and incubated for 30 min at 37 °C in the dark. Superoxide production was measured immediately by flow cytometry as an increase in fluorescence intensity (FL-2 channel).

### Ca^2+^ Flux

Platelet-rich plasma was loaded with the cell membrane permeable Ca^2+^-sensitive dye Fluo-3-AM (5 µM) in the presence of 2.5 mM probenecid for 30 min at 37 °C and resuspended in Tyrode’s buffer (1.5 µL of PRP/500 µL buffer). Changes in intracellular Ca^2+^ levels in platelets were measured by flow cytometry as an increase in fluorescence intensity of Fluo-3-AM in FL-1 channel.

### Phosphokinase array

Analysis of phosphorylation profiles of kinases and their protein substrates was done by the Human Phospho-Kinase Array (R&D Systems, Minneapolis, MN). Washed platelets were pretreated with nHDL (50 µg/mL) or sPLA_2_-HDL-high (50 µg/mL) for 5 min at RT, stimulated with collagen (5 µg/mL) or ADP (10 µM) for 5 min at RT and analyzed according to manufacturer’s protocol. The membranes were imaged using ChemiDoc Touch Imaging System and ECL Blotting Substrate (both Bio-Rad, Vienna, Austria). The average signal (pixel density) analysis of duplicate spots representing each protein was performed using Image Lab 5.2 software (Bio-Rad). A twofold increase/decrease in phosphorylation was considered to be significant.

### Western blot analysis

Washed platelets were pretreated with vehicle, nHDL (50 µg/mL), sPLA_2_-HDL-low (50 µg/mL) or sPLA_2_-HDL-high (50 µg/mL) for 5 min at RT and stimulated with either ADP (10 µM) or collagen (5 µg/mL) for 5 min at RT. Subsequently 6X Laemmli buffer containing 5% β-mercaptoethanol was added and samples were denaturated for 5 min at 95 °C. Equal amounts of protein were submitted to SDS-polyacrylamide gel electrophoresis (SDS/PAGE) and transferred to a polyvinylidene difluoride (PVDF) membrane. The membrane was cut into two parts in order to enable blotting for multiple antibodies. After blocking unspecific binding sites with a blocking buffer (137 NaCl, 20 mM Tris, 0.1% Tween-20. pH 7.6, with 5% milk powder), the upper part of the membrane was incubated with primary rabbit anti-phospho Akt antibody (1:1000) and the lower part with anti-phospho ERK1/2 antibody (1:1000) overnight at 4 °C. Membranes were rinsed and subsequently incubated with HRP-conjugated secondary Abs (1:5000) for 1 h at room temperature. Before probing with anti-ERK1/2 or anti-Akt primary antibodies (1:1000, overnight at 4 °C), membranes were stripped for 30 min at 50 °C using a stripping buffer (100 mM 2-mercaptoethanol, 2% SDS, 62.5 mM Tris-HCl, pH 6.8). As a loading control membranes were incubated with mouse anti-β-actin primary antibody (1:7500) overnight at 4 °C. ChemiDoc Touch Imaging System and ECL Blotting Substrate (both Bio-Rad, Vienna, Austria) were used to visualize protein bands. Immunoblot images were quantified using Image Lab 5.2 software (Bio-Rad).

### Statistical analysis

All data are shown as mean ± SEM for n separate experiments. Experiments were repeated three to six times using platelets from different donors. Statistical analyses were performed with GraphPad Prism Version 5. Comparisons of groups were performed using one-way ANOVA with Dunnett’s post-hoc test or two-way ANOVA with Bonferroni post-hoc test. Significances were accepted at *p < 0.05, **p < 0.01 and ***p < 0.001.

## Electronic supplementary material

Dataset 1
