# Supplementary material for: Secretory phospholipase A2 modified HDL rapidly and potently suppresses platelet activation
Source: Sci Rep. 2017 Aug 14;7:8030. doi: 10.1038/s41598-017-08136-1 (PMC5556053; doi:10.1038/s41598-017-08136-1)
Supplement: Supplementary file 1 — Dataset 1 [file 41598_2017_8136_MOESM1_ESM.doc]

**Supplementary Material**

**Secretory phospholipase A_2_ modified HDL rapidly and potently suppresses platelet activation**

Sanja Curcic^1^, PhD, Michael Holzer^1^, PhD, Lisa Pasterk^1^, MSc, Eva Knuplez^1^, MSc, Thomas O. Eichmann^2^, PhD, Saša Frank^3^, PhD, Robert Zimmermann^2^, PhD, Rudolf Schicho^1^, PhD, Akos Heinemann^1^, MD, and Gunther Marsche^1^,*, PhD.

^1^Institute of Experimental and Clinical Pharmacology, Medical University of Graz, Graz, Austria

^2^Institute of Molecular Biosciences, University of Graz, Graz, Austria

^3^Institute of Molecular Biology and Biochemistry, Medical University Graz, Graz, Austria

***Corresponding author:**

Gunther Marsche, PhD,

Institute of Experimental and Clinical Pharmacology

Medical University of Graz, Austria

Universitätsplatz 4, 8010 Graz

Tel.: +43 316 380 4513

Fax: +43 316 380 9645

E-mail: [gunther.marsche@medunigraz.at](mailto:gunther.marsche@medunigraz.at)

**Materials**

Annexin V was from BD Bioscience (Vienna, Austria) and HDL Purification Kit was from Cell Biolabs (San Diego, USA).

**Methods**

**Mass spectrometry analysis of lysophosphatidylcholines**

HDL samples (corresponding to 300 µg protein) were extracted twice according to Folch et al.^1^ using chloroform/methanol/water (2/1/0.6, v/v/v). Additionally, extraction solvents contained 500 nM butylated hydroxytoluene, 1% acetic acid, and 4 nmol internal standard (ISTD) mix (C17-LPC, C17-PC, C17-TAG; Avanti Polar Lipids, Alabaster, Alabama) per sample. Extraction was performed under constant shaking for 60 min at RT. After centrifugation at 1,000 x g for 15 min at RT the organic phase was collected. A second extraction was executed by the addition of chloroform to the residual phase with above described further proceedings. Combined organic phases of the double-extraction were dried under a stream of nitrogen and dissolved in 150 µL chloroform/methanol/2-propanol (2/1/12, v/v/v) for UPLC-qTOF analysis. Chromatographic separation was performed using an AQUITY-UPLC system (Waters Corporation), equipped with a HSS T3 column (2.1x100 mm, 1.8µm; Waters Corporation) as previously described^2^. A SYNAPT™G1 qTOF HD mass spectrometer (MS) (Waters Corporation, Milford, Massachusetts) equipped with an ESI source was used for detection. Data acquisition was done by the MassLynx 4.1 software (Waters Corporation). Lipid classes were analyzed with the “Lipid Data Analyzer 1.6.2” software^3^. Extraction efficacy and lipid recovery were normalized using ISTDs.

**Phosphatidylserine surface exposure**

To measure phosphatidylserine exposure to the surface of platelets, washed platelets were incubated for 15 min at RT with vehicle, HDL samples (50 µg/mL), sPLA_2_ (400 ng/mL) or varespladib (1 µM). Cells treated with ionophore A23187 (300 nM) were used as a positive control for apoptosis. To each sample 2.5 µL FITC labelled Annexin V was added and platelets were incubated for further 15 min at RT in the dark. Subsequently binding buffer was added according to the manufacturer’s protocol and phosphatidylserine exposure on platelets was measured immediately using flow cytometry.

**HDL isolation by dextran-sulfate precipitation**

For HDL isolation from plasma by dextran-sulfate precipitation, we used a commercially available kit from Cell Biolabs (HDL Purification Kit, Nr.: STA-607). The isolation was performed according to the manufacturer’s instructions. Control HDL from the same plasma donor was isolated first by ultracentrifugation and then precipitated with dextran-sulfate using the same HDL Purification Kit.

**Supplementary Figures**

**
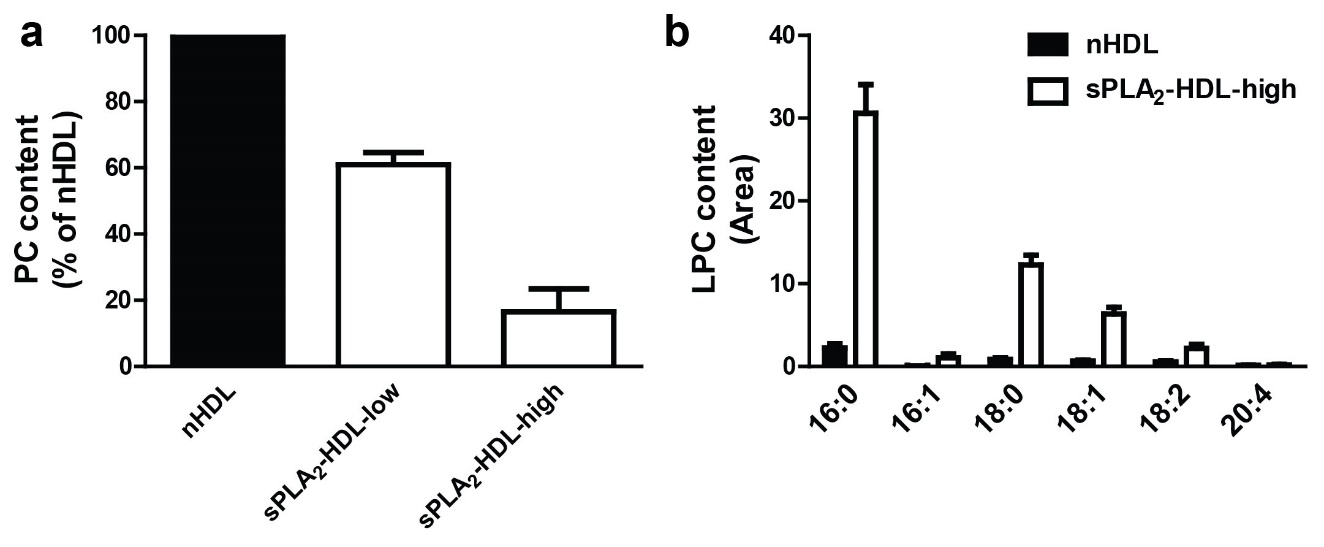
**

**Supplementary Figure 1. Analysis of LPC species present in sPLA_2_-HDL. (a)** Phosphatidylcholine (PC) content of native HDL (nHDL), sPLA_2_-HDL-low and sPLA_2_-HDL-high and **(b)** lysophosphatidylcholine (LPC) contents of nHDL and sPLA_2_-HDL-high were analyzed by mass spectrometry. Results are shown as mean ± SEM (n=4).

**
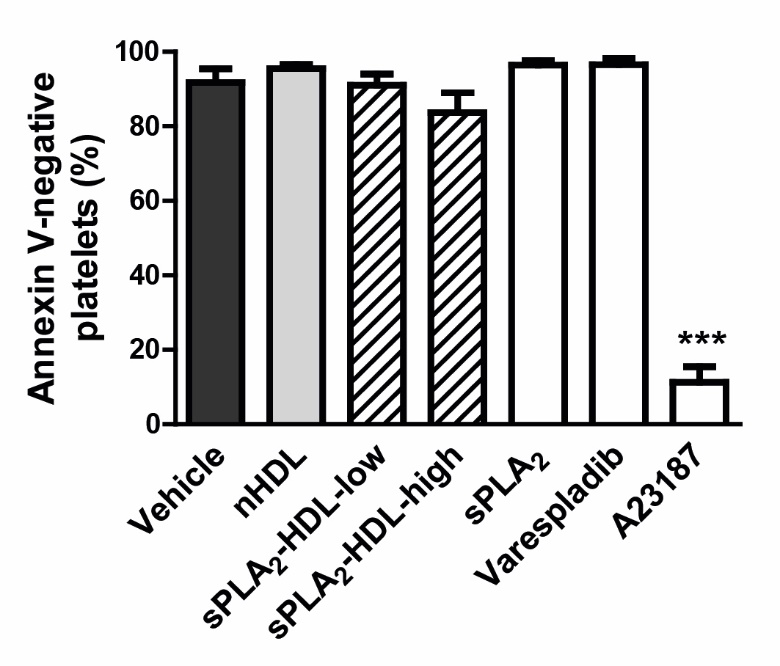
**

**Supplementary Figure 2. sPLA_2_-HDL does not induce phosphatidylserine expression in platelets.** Platelets were treated with vehicle, nHDL (50 µg/mL), sPLA_2_-HDL-low (50 µg/mL), sPLA_2_-HDL-high (50 µg /mL), sPLA_2_, varespladib or ionophore A23187 (300 nM). Phosphatidylserine surface expression was assessed by Annexin V staining by flow cytometry. Values are expressed as percentage of Annexin V-negative (viable) cells. Results are shown as mean ± SEM (n=3). Statistical significance was assessed by one-way ANOVA followed by Dunnett´s post hoc test. ***p < 0.001 versus vehicle-treated platelets.


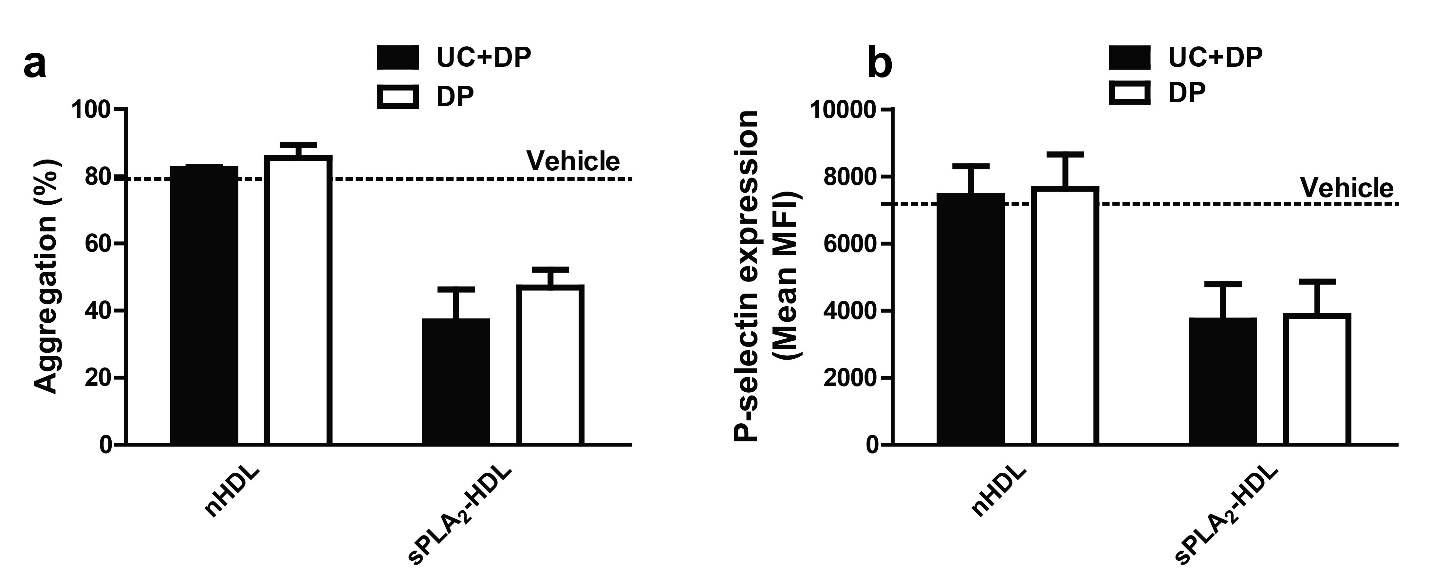


**Supplementary Figure 3. HDL isolation procedure does not influence the effects of sPLA_2_-HDL on platelets.** HDL was isolated from plasma using dextran-sulfate precipitation (DP). As a control, HDL from the same plasma was first isolated by ultracentrifugation and subsequently precipitated with dextran sulfate (UC+DP). UC+DP and DP isolated HDLs were treated with sPLA_2_ type V (overnight, 37ᵒC). Platelets were pretreated with either vehicle, nHDL or sPLA_2_-HDL (50 µg/mL). **(a)** Platelets were stimulated with ADP in concentrations which induced 70-90 % aggregation in vehicle-stimulated platelets. Values are expressed as % of maximal platelet aggregation. The dotted line represents aggregation of ADP-stimulated platelets in the absence of HDL. **(b)** Platelets were stimulated with ADP (3 µM) in the presence of cytochalasin B (5 µg/mL) and P-selectin expression was assessed by flow cytometry. The dotted line represents P-selectin expression in platelets stimulated with ADP and cytochalasin B in the absence of HDL. Results are shown as mean ± SEM (n=3). Statistical significance was assessed by two-way ANOVA followed by Bonferroni post hoc test.

***
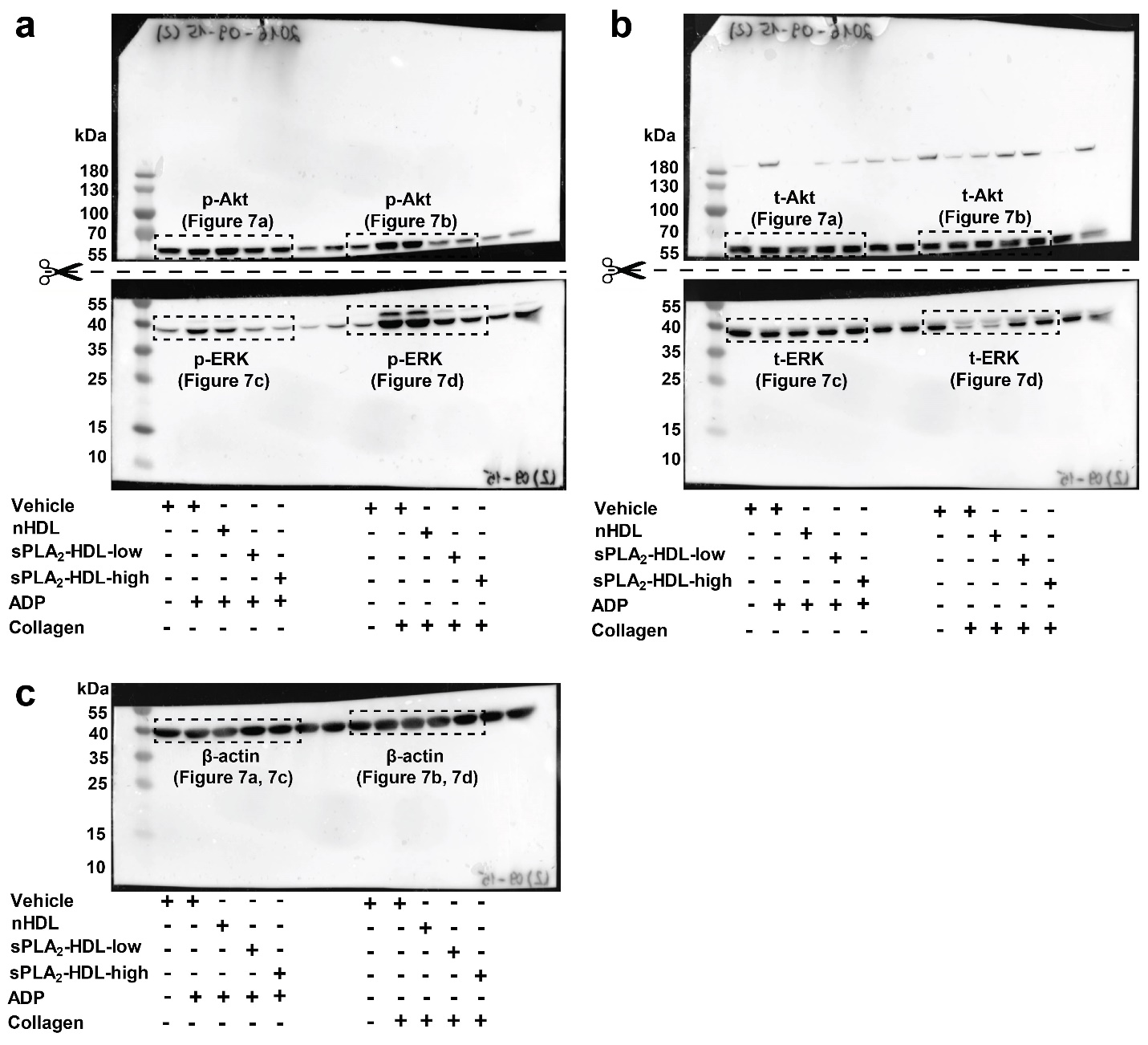
***

**Supplementary Figure 4. Full-length blots of p-Akt, t-Akt, p-ERK1/2, t-ERK1/2 and β-actin (shown as cropped images in Figure 7).** Platelets were pretreated with vehicle, nHDL (50 µg/mL), sPLA_2_-HDL-low (50 µg/mL) or sPLA_2_-HDL-high (50 µg/mL) and stimulated with ADP (10 µM) or collagen (5 µg/mL). Akt (Ser473) and ERK1/2 phosphorylation was assessed by Western blot. After transfer of the proteins to a PVDF-membrane, the membrane was cut into two parts. **(a)** The upper part of the membrane (containing proteins with a molecular weight larger than 55kDa) was probed with anti-phospho Akt antibody. The lower part of the membrane (containing proteins with a molecular weight lower than 55kDa) was probed with anti-phospho ERK antibody. **(b)** Membranes were stripped and reprobed for total Akt (the upper part of the membrane) and total ERK (the lower part of the membrane). **(c)** The lower part of the membrane was stripped again and, as a loading control, the levels of β-actin were detected using anti β-actin antibody. ChemiDoc Touch Imaging System and ECL Blotting Substrate (both Bio-Rad, Vienna, Austria) were used to visualize protein bands. Immunoblot images were quantified using Image Lab 5.2 software (Bio-Rad).

References

1. FOLCH, J., LEES, M. & SLOANE STANLEY, G. H. A simple method for the isolation and purification of total lipides from animal tissues. *J. Biol. Chem.* **226**, 497-509 (1957).

2. Knittelfelder, O. L., Weberhofer, B. P., Eichmann, T. O., Kohlwein, S. D. & Rechberger, G. N. A versatile ultra-high performance LC-MS method for lipid profiling. *J. Chromatogr. B. Analyt Technol. Biomed. Life. Sci.* **951-952**, 119-128 (2014).

3. Hartler, J. *et al*. Lipid Data Analyzer: unattended identification and quantitation of lipids in LC-MS data. *Bioinformatics* **27**, 572-577 (2011).
